# Pyrexia With Pancytopenia: An Uncommon Presentation of Kikuchi Disease

**DOI:** 10.7759/cureus.75588

**Published:** 2024-12-12

**Authors:** Esha Wijethilake, Yamuna Ranaweera, Dilan Dharshana Eleperuma, MI Mujahieth, Kamali Edirisinghe

**Affiliations:** 1 Internal Medicine, National Hospital of Sri Lanka, Colombo, LKA; 2 Otolaryngology - Head and Neck Surgery, National Hospital of Sri Lanka, Colombo, LKA

**Keywords:** cervical lymphadenopathy, kikuchi disease, necrotising lymphadenitis, pancytopenia, pyrexia of unknown origin

## Abstract

Kikuchi-Fujimoto disease is a rare systemic illness commonly affecting young females with a higher tendency to occur in the Asian population. Clinical presentation varies with most patients presenting with fever and cervical lymphadenopathy. The patient discussed in this case report presented to a tertiary care hospital in Sri Lanka with a fever for two weeks and palpable cervical lymphadenopathy. She was extensively evaluated as a case of pyrexia of unknown origin and failed to detect any significant abnormality except pancytopenia, which is a rarely associated hematological manifestation. The diagnosis was confirmed by cervical lymph node excision biopsy, which showed typical features to suggest Kikuchi disease. She was treated accordingly with steroids, resulting in a complete resolution of symptoms. This is a rare clinical entity that may give rise to diagnostic difficulty due to its rarity as well as the uncommon associations with pancytopenia. Further, though Kikuchi disease is commonly self-limiting, it may mimic many sinister pathologies, which warrant their exclusion.

## Introduction

Kikuchi disease, also known as Kikuchi-Fujimoto disease (KFD) or histiocytic necrotizing lymphadenitis, is a rare benign systemic disorder commonly affecting young Asian females. A patient with KFD will present with fever and cervical or generalized lymphadenopathy. This may further be associated with nausea, weight loss, night sweats, arthralgia, or hepatosplenomegaly [1]. The clinical picture can be complicated with hematological alterations, though mostly, the complete blood count is normal. The abnormality may be either leukopenia (up to 43% in early studies) or leukocytosis [2].

This was first described by Kikuchi and Fujimoto in 1972 [3,4] in young Japanese women, of which the exact incidence is unknown [5]. Existing data suggest females are more commonly affected with a female-to-male ratio of 2:1 [5]. The disease is mostly described in the Asian population, especially between the ages of 20 and 35 years, than in the Western population [5,6]. The association of pancytopenia is relatively rare, with a quoted frequency of 2% [6]. Other commoner hematological manifestations are anemia, lymphopenia, neutropenia, thrombocytopenia, and the presence of atypical lymphocytes [6]. Histopathology plays a vital role in making accurate diagnoses, as Kikuchi disease can mimic sinister diseases like tuberculosis, lymphoma, and metastatic malignancy [7].

Here, we present a case of a young female who presented with fever and associated pancytopenia, whose diagnosis was confirmed as Kikuchi disease.

## Case presentation

A 32-year-old female patient with a past history of post-streptococcal glomerulonephritis presented with high-grade fever associated with arthralgia, myalgia, increased fatiguability, and malaise for a duration of two weeks. She had a significant loss of appetite and loss of weight as well. She did not reveal a past history of oral ulcers, alopecia, photosensitive rashes, any other cutaneous manifestations, or inflammatory-type arthritis. There was no history of chronic cough, hemoptysis, or past history of or close contact history of tuberculosis. She denied recent travel history, risky sexual encounters, intravenous drug abuse, or blood transfusions.

Examination revealed that the patient was febrile and pale, with a body mass index of 23 kg/m^2^. There was non-tender cervical lymphadenopathy, with the largest being 2 cm in diameter in the left side level five lymph node group. Associated palpable inguinal or axillary lymphadenopathy was not there. There was no alopecia, peri-orbital puffiness, or ankle edema.

The cardiovascular system revealed blood pressure of 110/70 mmHg with a pulse rate of 100 bpm, which was of normal volume. She maintained the oxygen saturation at 98% in room air, with bilateral air entry being equal without added sounds. There was a palpable spleen up to 2 cm below the costal margin without associated hepatomegaly or free fluid in the abdomen. Neurological examination revealed no abnormality.

Full blood count on admission showed a total white cell count of 1.35 × 10^9^ cells/L, hemoglobin 10 g/dL, and platelet count of 84 × 10^9^ cells/L. The blood picture revealed features that suggested acute viral infection or an autoimmune process. Her erythrocyte sedimentation rate was 75 mm at the first hour, while C-reactive protein was 35 mg/dL.

**Figure 1:**
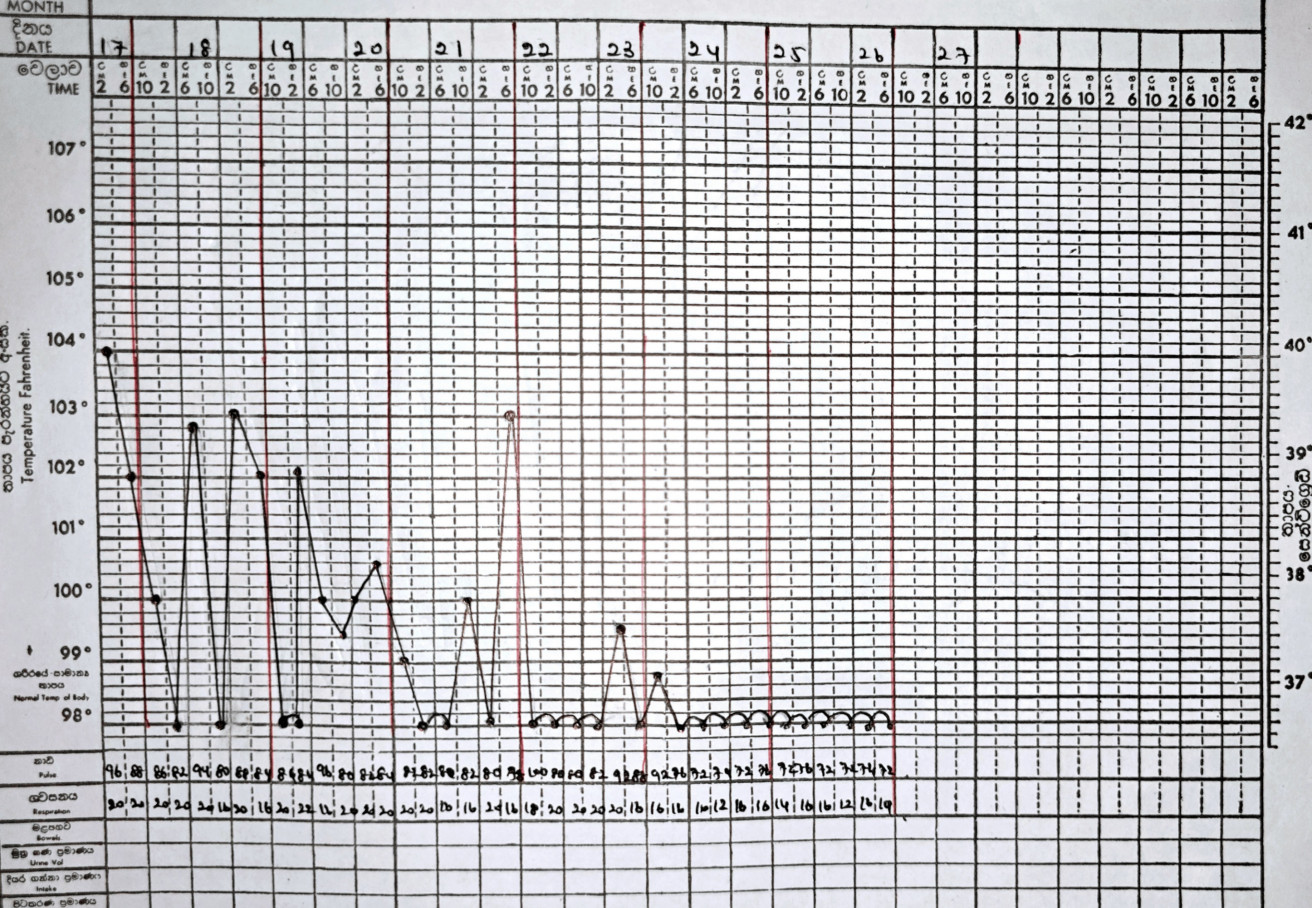
Temperature chart showing trend of fever pattern

With the above presentation, she was initially treated for an infective disease with the commencement of broad-spectrum intravenous antibiotics and reversed barrier nursing care. Yet her fever did not show any response. Her fever chart, indicating the trend, is depicted in Figure 1.

Investigations were conducted to confirm or refute any infective, autoimmune, or malignant pathology. These are summarized in the tables below. Hematological parameters with their relative trend are given in Table 1, while biochemical evaluations are depicted in Table 2. Table 3 gives a snapshot of the microbiological investigations. Table 4 illustrates the immunology panel.

**Table 1:** Hematological investigations and trend

Investigation	Reference	Day one	Day three	Day five
White blood cell count	4.5-11.0 × 10^9^/L	1.35 × 10^9^/L	0.84 × 10^9^/L	0.66 × 10^9^/L
Neutrophils	1.5-8.0 × 10^9^/L	0.92 × 10^9^/L	0.52 × 10^9^/L	0.29 × 10^9^/L
Lymphocytes	1.0-4.8 × 10^9^/L	0.2 × 10^9^/L	0.26 × 10^9^/L	0.32 × 10^9^/L
Eosinophils	0.02-0.5 × 10^9^/L	0	0	0
Hemoglobin	12-15.5 g/dL	10 g/dL	7.5 g/dL	11.3 g/dL
Mean corpuscular volume	80-100 fL	80 fL	84 fL	73 fL
Platelet count	150-450 × 10^9^/L	84 × 10^9^/L	60 × 10^9^/L	73 × 10^9^/L
International normalized ratio (INR)	0.8-1.1	0.8	0.9	0.8
Activated partial thromboplastin time	21-35 seconds	28 seconds	30 seconds	29 seconds
Bleeding time	One to nine minutes	Two minutes	Three minutes	Two minutes

**Table 2:** Biochemical investigations

Investigation	Value	Reference range
Serum ferritin	2150 ng/mL	13-150 ng/mL
Lactate dehydrogenase level	250 U/L	140-280 U/L
Procalcitonin	0.76 ng/mL	<0.05 ng/mL
Serum triglyceride level	237 mg/dL	<150 mg/dL
Urinary protein creatinine ratio	0.1 mg/mg	<0.2 mg/mg
Urinary dysmorphic red cells	Not seen	<2%
Serum fibrinogen	2.4 g/L	2-4 g/L
Serum creatinine	0.6 mg/dL	0.6-1.1 mg/dL
Serum sodium	137 mmol/L	136-145 mmol/L
Serum potassium	3.7 mmol/L	3.5-5.2 mmol/L
Blood urea	17 mg/dL	6-21 mg/dL
Serum corrected calcium	8.9 mg/dL	8.5-10.2 mg/dL
Serum phosphate	3.6 mg/dL	2.5-4.5 mg/dL
Serum uric acid	4 mg/dL	3.5-7.2 mg/dL
Alanine transaminase	35 U/L	19-25 U/L
Aspartate transaminase	28 U/L	10-36 U/L
Serum total bilirubin	0.5 mg/dL	0.1-1.2 mg/dL
Serum direct bilirubin	0.1 mg/dL	<0.3 mg/dL
Serum albumin	3.1 g/dL	3.4-5.4 g/dL
Serum globulin	3.1 g/dL	2-3.5 g/dL
Serum alkaline phosphatase	40 U/L	30-130 U/L
Serum gamma-glutamyl transferase	22 U/L	5-40 U/L
Serum angiotensin-converting enzyme level	<40 nmol/mL/minute	<40 nmol/mL/minute

**Table 3:** Microbiological investigations

Bacterial investigations		Viral investigations	
Three peripheral blood cultures	Negative	Dengue NS-1 antigen	Negative
Mantoux test	Negative	Dengue IgM antibodies	Negative
Sputum culture	Negative	Dengue IgG antibodies	Negative
Urine culture	Negative	Epstein-Barr IgM antibodies	Negative
Sputum acid fast bacilli	Negative	Cytomegalovirus IgM antibodies	Negative
Mycoplasma antibodies	Negative	Hepatitis B surface antigen and antibodies	Negative
Bone marrow tuberculosis PCR	Negative	Hepatitis C antibodies	Negative
Melioidosis antibodies and culture	Negative	HIV 1 and HIV 2 antigens and antibodies	Negative
Rickettsial antibodies	Negative	Fungal cultures	Negative
Leptospirosis antibodies	Negative	-	-
Toxoplasma antibodies	Negative	-	-

**Table 4:** Immunology panel

Investigation	Result
Anti-nuclear antibodies (ANA)	Negative
Double-stranded DNA (DsDNA)	Negative
Anti-Jo-1 antibodies	Negative
Ribonucleoprotein antibodies	Negative
Anti-Smith antibodies	Negative
Anti-PM1 scleroderma antibodies	Negative
Anti-Ro antibodies	Negative
Anti-La antibodies	Negative
Serum C3 level (mg/dL)	127 (83-177)
Serum C4 level (mg/dL)	33 (12-36)

Considering all the blood investigations, the patient did not have any major abnormality except pancytopenia. Her biochemical panel reveals increased serum ferritin and procalcitonin, indicating an inflammatory process. All her microbiological and immunological investigations were normal, excluding a septic focus for pyrexia and any serious autoimmune disorder.

**Figure 2:**
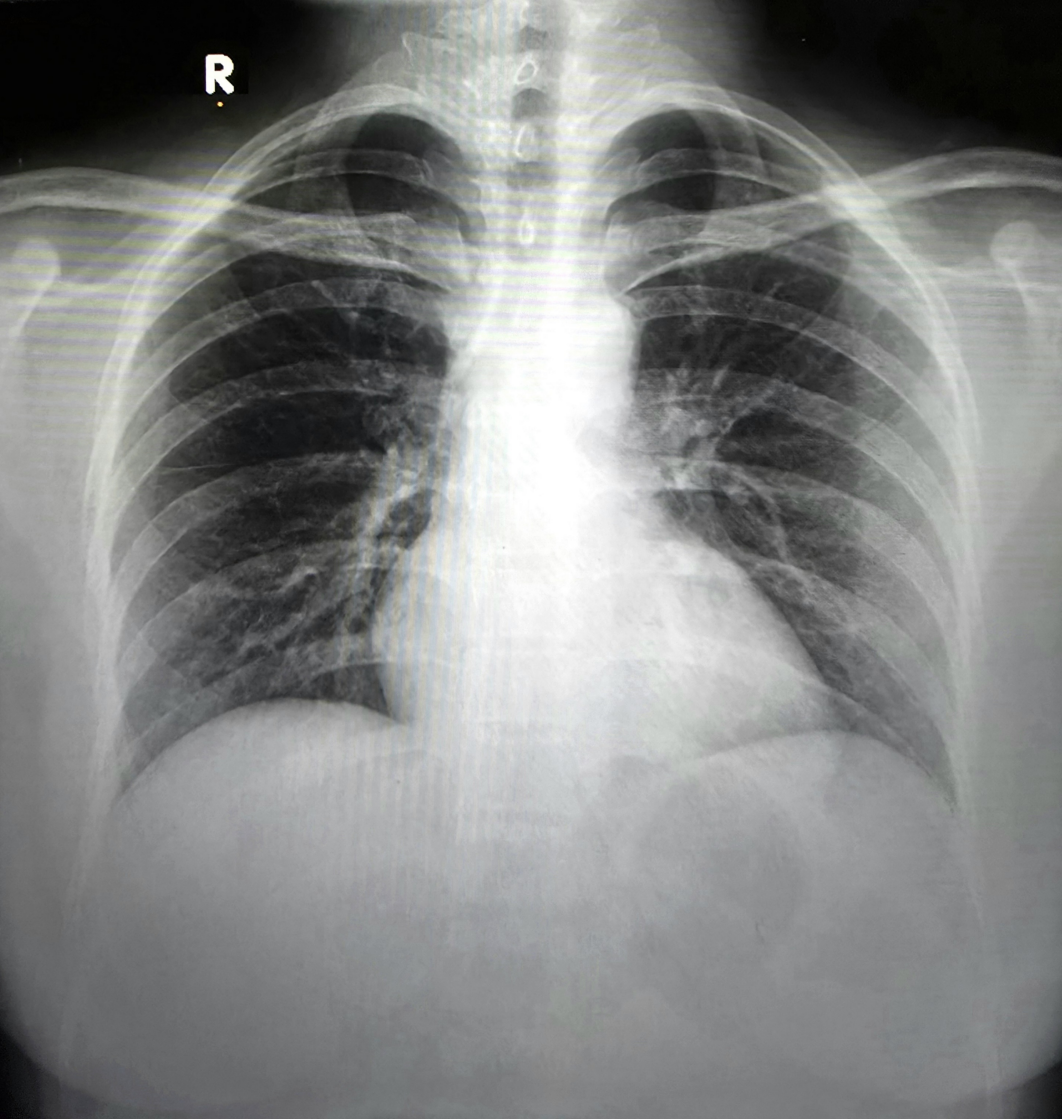
Chest X-ray postero-anterior view

The patient underwent imaging studies to evaluate causes for pyrexia with chest X-ray (given in Figure 2), ultrasonography, and contrast-enhanced CT (CECT) of the chest, abdomen, and pelvis. The chest X-ray did not reveal any abnormality. The ultrasound scan (USS) of the abdomen revealed a grade one fatty liver and mild splenomegaly (spleen 13 cm), while there were no ascites or pleural effusion nor any intra-abdominal lymphadenopathy. The CECT revealed bilateral axillary lymphadenopathy and mild splenomegaly.

Her bone marrow trephine biopsy showed mildly hypercellular marrow showing suppressed erythropoiesis with maturation arrest and normal megakaryopoiesis. Findings were in favor of an acute viral infection or causing transient bone marrow suppression leading to pancytopenia. No clear evidence of hemophagocytic lymphohistiocytosis. There was no evidence of malignant disease infiltrating bone marrow. Bone marrow bacterial culture was negative.

**Figure 3:**
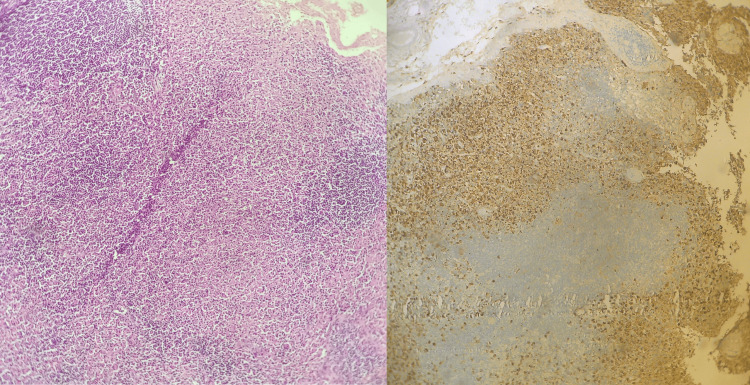
Histopathological images of hematoxylin and eosin (H&E) staining (left) showing necrotizing lymph node (H&E, 10×) and immunohistochemistry (IHC) for myeloperoxidase (right) showing positivity in the histiocytes (IHC, 10×)

The patient underwent an excision biopsy of the palpable cervical lymph node in the left posterior triangle. Grossly, the lymph node measured 12 × 10 mm. Histopathology of the lymph node showed partially effaced architecture. The paracortex, cortex, and medulla were expanded by abundant karyorrhectic debris, eosinophilic granular material, and histiocytes. Intact neutrophils were absent. Granulomas or atypical lymphocytes were not seen. No extranodal extension. Immunohistochemistry for myeloperoxidase showed strong cytoplasmic granular positivity in many of the histiocytes. The histopathological diagnosis of the right cervical lymph node was that of necrotizing lymphadenitis consistent with KFD (Figure 3).

Once the diagnosis has been confirmed by lymph node biopsy as KFD, the management will commence. Accordingly, the patient was administered steroids according to recommendations. An intravenous methylprednisolone pulse of 500 mg was administered daily for three days. This management gave the patient a significant improvement in the resolution of fever and neutropenia.

During the follow-up assessment, the patient had no palpable lymphadenopathy. She underwent an ultrasound of the neck and abdomen, which showed a resolution of cervical lymphadenopathy and splenomegaly.

## Discussion

KFD is a self-limiting benign condition that commonly affects young females, mainly of Asian ethnicity. The incidence of Kikuchi disease is unknown [5].

The etiopathogenesis of the disease is unclear. However, it has been suggested that it occurs as a hyperimmune response to an infectious agent. Epstein-Barr virus was the most identified pathogen [7]. Other postulations state it may be an auto-immune disorder. Other infectious agents include cytomegalovirus, human herpesvirus-7, parvovirus B19, and mycobacterial species [5,8].

This is a systemic illness that presents with prolonged fever, lymphadenopathy (commonly cervical, may have generalized involvement), rash (maculopapular), fatigue, arthritis, and hepatosplenomegaly [5]. Other less common clinical manifestations include weight loss, night sweats, nausea, vomiting, and diarrhea.

A study done among 282 patients of KFD showed hematological abnormalities, including anemia (22%), lymphopenia (17%), neutropenia (11%), atypical lymphocytes (9%), and thrombocytopenia (8%) [6]. Bicytopenia (5%) and pancytopenia (2%) have been uncommon manifestations [6]. Here, the index patient presented with prolonged fever, lymphadenopathy, splenomegaly, and pancytopenia. Pancytopenia has varied etiologies ranging from infections like tuberculosis, malignancies like lymphomas, and autoimmune diseases like systemic lupus erythematosus (SLE) [9]. The differential diagnoses entertained were infectious mononucleosis, tuberculosis, lymphoma, and SLE.

Since the clinical presentation mimics sinister diseases, misdiagnosis occurs in about 40% of patients [7]. Diagnosing Kikuchi disease could be challenging as it displays nonspecific clinical presentations. Accurate diagnosis of Kikuchi disease can be demonstrated by histological examination of a lymph node. Findings of this disease could be difficult to differentiate from SLE [7]. There have been instances in some patients who developed SLE later [10]. Literature also reveals instances where Kikuchi disease coexisted with SLE or even followed by the development of SLE [10]. If Kikuchi coexists with SLE, it has been reported to have a much more aggressive disease course.

Typical histological findings include preserved nodal architecture with abundant karyorrhectic debris, coagulative necrosis in paracortical areas, and an absence of eosinophils or neutrophils [7]. Our patient’s histology showed abundant karyorrhectic debris, eosinophilic granular material, and histiocytes, but no intact neutrophils were present. Also, her antibody panel was negative for SLE. Serological tests to exclude infectious diseases also play an important role in arriving at the final diagnosis.

Imaging, including USS and CECT, would support the diagnosis by excluding differential diagnoses like lymphoma and tuberculosis. CECT of the lymph nodes illustrates perinodal infiltration and homogenous contrast enhancement.

There is no universally approved treatment protocol for Kikuchi disease [11]. Patients are usually treated symptomatically once the sinister pathologies have been excluded. Symptoms resolve spontaneously within one to three months [11]. Systemic glucocorticoids [12] or high-dose glucocorticoids with immunoglobulin [13] have been shown to be beneficial when the patients develop severe manifestations or if the symptoms do not resolve spontaneously. Our patient markedly improved with the commencement of steroids, with significant improvement in her fever and cytopenia, while the follow-up assessment further revealed the complete resolution of the disease.

## Conclusions

This case report highlights the thorough clinical evaluation and the necessary investigations for the timely diagnosis of Kikuchi disease. Although it is a benign condition, the correct diagnosis will be beneficial to commence the necessary treatment at the correct time. It is always advisable to exclude associated autoimmune diseases like SLE, especially when the patient presents with uncommon manifestations such as pancytopenia. Further, the patients detected to have Kikuchi disease have a tendency to develop systemic lupus erythematosus later in life. Therefore, long-term follow-up of these patients is recommended.
